# Information encoding and transmission profiles of first-language (L1) and second-language (L2) speech*

**DOI:** 10.1017/s1366728921000717

**Published:** 2021-08-18

**Authors:** Ann R. Bradlow

**Affiliations:** Department of Linguistics, Northwestern University, Evanston, Illinois, USA

**Keywords:** second-language speech production, speech rate, communication efficiency, information density

## Abstract

Inspired by information theoretic analyses of L1 speech and language, this study proposes that L1 and L2 speech exhibit distinct information encoding and transmission profiles in the temporal domain. Both the number and average duration of acoustic syllables (i.e., intensity peaks in the temporal envelope) were automatically measured from L1 and L2 recordings of standard texts in English, French, and Spanish. Across languages, L2 acoustic syllables were greater in number (more acoustic syllables/text) and longer in duration (fewer acoustic syllables/second). While substantial syllable reduction (fewer acoustic than orthographic syllables) was evident in both L1 and L2 speech, L2 speech generally exhibited less syllable reduction, resulting in low information density (more syllables with less information/syllable). Low L2 information density compounded low L2 speech rate yielding very low L2 information transmission rate (i.e., less information/second). Overall, this cross-language comparison establishes low information transmission rate as a language-general, distinguishing feature of L2 speech.

## Introduction

When individuals know two or more languages, variation in the onset and extent of exposure to each language typically results in a functional imbalance across the first-language (L1), learned from early and extended exposure, and the second language (L2), learned from later and more limited exposure^1^. This experience-dependent L1-L2 imbalance is evident in L1 versus L2 speech patterns. Just as listeners can identify individual talkers (e.g., Schweinberger & Zaske, 2018) and languages (e.g., Vaughn & Bradlow, 2017) from short speech samples, listeners can also determine whether an utterance was produced in L1 or L2 speech (i.e., sounds “unaccented” or “foreign-accented”) from short snippets of speech in a known language (Flege, 1984; Munro, Derwing & Burgess, 2003, 2010) and even in an unknown language (Bond, Stockmal & Markus, 2003, 2008; Major, 2007). These observations suggest patterns of speech production associated with an L1 or L2 speech “mode” regardless of the language being spoken or of language-specific L1-L2 interactions.

The present study seeks to identify language-general acoustic properties that distinguish L1 and L2 speech and that may underlie the observed rapid identification of speech samples as either L1 or L2 speech. Furthermore, inspired by information theoretic analyses of L1 speech and language production, this study proposes that L1 and L2 speech exhibit distinct information encoding and transmission profiles in the temporal domain. Accordingly, the present study compares speech timing patterns in L1 and L2 speech in three languages: English, French, and Spanish. This is done in terms of two key information theoretic parameters: information density (number of speech units for a given meaning) and information transmission rate (information conveyed per second). Identification of a language-general, information-driven temporal parameter along which L1 and L2 speech differ would broaden our perspective on the communicative impact of speech communication between speakers from different language backgrounds to include the impact of L1 versus L2 speech mode on the dynamics of information flow via the speech channel. This study thus represents an initial step towards characterizing the language-general L1 versus L2 speech mode in quantifiable information theoretic terms that can be automatically applied to a multi-lingual multi-talker speech corpus. This empirical base can then support further speculation and theorizing regarding cognitive mechanisms that underlie L1 versus L2 speech production and their impact on communicative efficiency (i.e., beyond speech intelligibility) under a variety of conversational conditions.

Prior work on information encoding and transmission in language and speech supports two inter-connected claims: (1) variation in complexity at all levels of linguistic structure is related to variation in predictability given the context (i.e., conditional entropy, or surprisal/redundancy), and (2) the distribution of information across utterances tends toward uniform density of encoding (i.e., constant degree of surprisal/redundancy) in order to optimize information transmission via the capacity-limited speech channel (Aylett & Turk, 2004; Crocker, Demberg & Teich, 2016; Jaeger, 2010; Jaeger & Tily, 2011; Levy, 2008). This information theoretic perspective on language and speech has provided insightful accounts of intra-language variation. For example, in order to adhere to a principle of “uniform information density” (Jaeger, 2010; Jaeger & Tily, 2011; Levy, 2008) or “smooth signal redundancy” (Aylett & Turk, 2004, 2006; Turk, 2010), syntactic and phonetic reduction phenomena – a major source of intra-language variation – abound when surprisal is low (i.e., when redundancy, or predictability, is high – see also Bell, Brenier, Gregory, Girand & Jurafsky, 2009; Cohen Priva, 2015; Gahl, Yao & Johnson, 2012; Johnson, 2004; Jurafsky, Bell, Gregory & Raymond, 2001 and many others).

A related line of research operationalized information density for cross-language comparisons in terms of the number of linguistic units over which a given meaning, or text, is distributed (Coupé, Oh, Dediu & Pellegrino, 2019; Fenk-Oczlon & Fenk, 2010; Pellegrino, Coupé & Marsico, 2011). This research proposed the syllable as an appropriate unit for cross-language comparison based on its universal applicability – all languages have a unit that is comprised of a sequence of segments even if the phonological status of this unit varies – and its quantifiability in terms of intensity peaks in the speech signal (acoustic syllables) or by metalinguistic counts from first-language speakers (phonological syllables). Languages that express a given meaning (i.e., direct translations of a given text) in relatively few syllables have higher syllable information density (more information conveyed per syllable) than languages that express the same meaning in relatively many syllables. Importantly, these cross-language comparisons have revealed a trade-off between speech rate and information density such that languages with relatively low information density (few syllables for a given meaning) exhibit relatively high speech rates (many syllables per second), and vice versa. Thus, while languages vary substantially in syllabic speech rate, the rate of information transmission (information conveyed per second) is more constrained across languages (Pellegrino et al., 2011; Coupé et al., 2019). Together, the intra-language tendency toward uniform information density (or, smooth signal redundancy) through suprisal-modulated complexity (or, redundancy-induced reduction), and the inter-language constraint on information rate through a trade-off between syllable rate and syllable information density provide converging evidence for optimization of information encoding and transmission as a universal tendency in speech and language production.

Given that a defining difference between L1 and L2 speech is variation in experience with the language, we might expect significant differences across L1 and L2 speech in surprisal/redundancy-driven modulation of complexity. L1 talkers have deeply entrenched implicit knowledge of the statistical structure of the language including the conditional probabilities of linguistic units at all levels (i.e., from phonotactic-level to syntactic-level probabilities). L1 talkers therefore exhibit reduction patterns that are finely tuned to the contours of surprisal (or conversely, redundancy) during L1 speech production (e.g., see Brandt, 2019 for an extensive study of suprisal and phonetic reduction). Specifically, L1 speakers systematically increase information density through phonetic reduction (same meaning distributed over less phonetic material) precisely where contextually determined suprisal is low (or redundancy is high – see also Aylett & Turk, 2004, 2006; Turk, 2010). In contrast, the language models of L2 talkers likely developed on the basis of explicit instruction about canonical grammatical structures, including dictionary-based pronunciations of words, rather than on early and continuous experience with naturally occurring conversational speech. Furthermore, the language model that underlies L2 speech production will be influenced by interactions between the L1 and L2 sound structures, including the size and structure of their phoneme inventories, their characteristic patterns of phoneme combination (i.e., the phonotactics), and their prosodic structures. Thus, as a consequence of the different L1 and L2 learning contexts and the L1-L2 interactions that influence L2 speech, the language models of L1 and L2 talkers may diverge substantially, leading to different estimations of suprisal/redundancy for particular words and phonemes in a given context, and thus to different patterns of context-dependent reduction. Regardless of whether surprisal/redundancy-driven phonetic reduction is primarily talker-oriented (i.e., arises from unit selection and production planning processes), listener-oriented (i.e., serves to ensure accurate communication) or driven by general evolutionary dynamics^2^, this view predicts different L1 and L2 patterns of phonetic reduction. While phonetic reduction is evident to some extent in both L2 and L1 speech (e.g., Schertz & Ernestus, 2014) and particularly when related to repetition within a discourse (e.g., Gustafson & Goldrick, 2018), where direct comparisons have been performed, L2 speech is generally more conservative in this respect than L1 speech (e.g., Baker, Baese-Berk, Bonnasse-Gahot, Kim, Van Engen & Bradlow, 2011; Spilková, 2014; Rallo Fabra, 2015; Oh & Lee, 2016; Li, Li, Luo & Mok, 2018). For example, Baker et al. (2011) found that L2 talkers exhibited less phonetic reduction than L1 talkers for segments in function words relative to in content words. Similarly, Spilková (2014), Oh and Lee (2016), and Li et al. (2018) all found that both L1 and L2 talkers of English exhibited predictability-related phonetic reduction – however, the degree of reduction was smaller for the L2 English talkers, particularly those with lower L2 proficiency, than for the L1 talkers; and Rallo Fabra (2015) found less extensive unstressed vowel reduction in English for early and late Spanish-English bilinguals as compared to English monolinguals. This pattern of less phonetic reduction in L2 than in L1 speech results in overall lower information density for L2 speech than for L1 speech since, for any given text (i.e., meaning), the L2 production involves more phonetic content than the L1 production.

Another salient, language-general difference between L1 and L2 speech is overall speech rate. Slower speaking rates (fewer syllables or words per second) for L2 versus L1 speech have been shown for L2 speech in various languages, including English (Guion, Flege, Liu & Yeni-Komshian, 2000; Baese-Berk & Morrill, 2015), Spanish (Garcia Lecumberi, Cooke & Wester, 2017), Japanese (Idemaru, Gubbins & Wei, 2019), and Dutch (De Jong, Groenhout, Schoonen & Hulstijn, 2015). Slower L2 speech rates have also been shown for between-language comparisons within individuals (e.g., Towell & Dewaele, 2005; Derwing, Munro, Thomson & Rossiter, 2009; De Jong et al., 2015; Bradlow, Blasingame & Kim, 2017). Taken together, this research establishes speech rate as a phonetic parameter that is strongly influenced by L1 versus L2 mode independently of the language(s) involved.

Both speech rate and phonetic reduction influence the flow of linguistic information as the speech signal unfolds in time. For a given utterance a slower speech rate necessarily implies a slower information transmission rate (fewer bits of information conveyed per unit of time). Similarly, since less phonetic reduction implies production of more phonetic material and since all phonetic material has some acoustic duration, the tendency of L2 speech to involve fully articulated instead of reduced forms also effectively decreases the rate of information transmission for L2 relative to L1 speech. Thus, the characteristically slow rate of L2 speech presumably combines with the tendency of L2 speech towards fully articulated rather than phonetically reduced forms (i.e., low information density) to produce an L2 speech mode with a very low information transmission rate. Importantly, this low L2 information transmission rate results from a combination of slow speaking rate and low information density, a combination that is contrary to the efficiency-driven trade-off between speech reduction and information density. The present study explores this information theoretic perspective on L2 speech through a close examination of syllable rate and syllable information density – two global speech properties that together influence information transmission rate – in L1 and L2 speech across three languages.

English, French, and Spanish are well-suited to this multi-language analysis because all three are widely spoken as both L1 and L2 and differ notably in their sound structures. In the World Atlas of Languages Structures Online (WALS, Dryer & Haspelmath, 2013), English and French are listed with extreme values for each of three critical phonological features: segment inventory size (both have large inventories), consonant-to-vowel ratio (both have low ratios), and complex syllable structures (both have complex syllable structures). In contrast, Spanish is listed with the central value on the WALS 3- or 5-point scales for each of these phonological features: average segment inventory, average consonant-to-vowel ratio, and moderately complex syllable structure (Maddieson, 2013a, 2013b, 2013c, 2013d; Goedemans & van der Hulst, 2013). In addition, English, French, and Spanish each exhibit several contextually conditioned phonological processes that affect timing patterns in connected speech. These include, amongst many others, unstressed vowel reduction in English, liaison in French, and consonant lenition in Spanish. While identification of the phonetic and perceptual correlates of isochrony is debated in the phonetics and psycholinguistics literature, it is note-worthy that the traditional rhythm-class hypothesis (Pike, 1945; Abercrombie, 1967) classifies English as stress-timed while French and Spanish are syllable-timed. Finally, downloadable recordings of a standard reading passage (the North Wind and Sun passage) in both L1 and L2 speech were available for all three languages.

To recap, this study aims to identify a language-general acoustic signature of the contrast between L1 and L2 modes of speech production in terms of information encoding and transmission in the temporal-domain. Through a novel application of information theoretic concepts to both L1 and L2 speech in three languages, this study represents an initial step towards a broader goal of gaining new insights into underlying mechanisms and communicative consequences of L1 versus L2 speech production. With the empirical contours of information transmission via the L2 speech channel in sharper focus, we will be poised for further speculation and hypothesizing for this broader research agenda.

### Methods and materials

The dataset for the present study consisted of 351 observations of 21 variables. Each observation is based on a digital speech recording of a given text by a given talker, and the variables are derived from automatic phonetic measurements applied to each recording. The digital speech recordings were taken from two separate speech corpora, the Northwestern University ALLSSTAR Corpus (Bradlow, n.d.; described in detail in Bradlow et al., 2017 and freely available to the public at https://speechbox.linguistics.northwestern.edu/#!/?goto=allsstar) (Bradlow, n.d.) and the University of Toronto Romance Phonetics Database (Colantoni & Steele, 2004, freely available to the public at http://rpd.chass.utoronto.ca/). The complete dataset and analysis scripts (R and Praat) are freely available from the Open Science Foundation (OSF) web-based repository via the following link: https://osf.io/vawdb/.

An overview of the 351 digital speech recordings included in this dataset is shown in Table 1. A total of 256 English recordings came from the ALLSSTAR Corpus. These English recordings consisted of readings of two texts by each of 128 talkers, the North Wind and the Sun (NWS) passage and a set of longer, complex sentences taken from the Universal Declaration of Human Rights (DHR). These 128 talkers included all of the L1 (n = 26) and L2 (n = 98) English talkers for whom English NWS and a complete set of English DHR recordings were available. All talkers were recruited from the Northwestern University community. The L2 talkers were mostly international graduate students. A small number of L2 talkers were family members of international students. English proficiency levels based on test scores (The Versant™ English Test, n.d.) were available for most (73/98 or 75%) of the L2 English talkers and showed a concentration at the intermediate level (43 talkers with Versant scores of 47–68 / 80) and advanced/near-native level (29 talkers with Versant scores of 69–80 / 80). Only one L2 English talker for whom proficiency information was available was at the beginner level (Versant score < 47 / 80). The L2 talkers came from 21 different L1 backgrounds distributed as follows: Cantonese (n = 11), Mainland Mandarin, (n = 13), Turkish (n = 13), Korean (n = 11), Spanish (n = 11), Hindi (n = 5), Brazilian Portuguese (n = 5), Russian (n = 5), Hebrew (n = 4), Vietnamese (n = 4), Farsi(Persian) (n = 3), Japanese (n = 3), German (n = 2), Singaporean Mandarin (n = 1), Taiwanese Mandarin (n = 1), French (n = 1), Gishu (n = 1), Greek (n = 1), Gujarati (n = 1), Indonesian (n = 1), and Runyankore (n = 1). The 26 L1 English talkers (14 females) had an average age of 20 years, and the 98 L2 English talkers (36 females) had an average age of 25 years at the time of recording.

Recordings from 103 talkers (61 recordings in French + 42 recordings in Spanish) came from the Romance Phonetics Database (RPD, http://rpd.chass.utoronto.ca/, Colantoni & Steele, 2004), an on-line research and teaching tool that includes recordings of individual words and passages in several Romance Languages (French, Italian, Portuguese, Romanian, and Spanish). Included in this database is a set of recordings of the NWS passage in L1 French (n = 14), L2 French (n = 47), L1 Spanish (n = 19) and L2 Spanish (n = 23). The RPD also includes a small number of NWS recordings in Italian, Portuguese, and Romanian; however, these were not included in the present study due to too few recordings in L2 speech (4 in L2 Italian, 0 in L2 Portuguese, and 3 in L2 Romanian) for meaningful comparison with either their L1 counterparts (2 in L1 Italian, 1 in L1 Portuguese, and 5 in L1 Romanian) or with other L2s included in the RPD (47 talkers in L2 French and 23 talkers in L2 Spanish) or with L2 English from the ALLSTAR Corpus (98 talkers).

The 14 L1 French (8 females) and 19 L1 Spanish (13 females) talkers had average ages of 31 and 29 years, respectively. The 47 L2 French (37 females) and 23 L2 Spanish (10 females) talkers had average ages of 27 and 31 years, respectively. The L2 French talkers came from 7 different L1 backgrounds distributed as follows: Albanian (n = 1), Arabic (n = 1), Mainland Mandarin (n = 3), Czech (n = 5), English (n = 31), Russian (n = 3), and Spanish (n = 3). Of the L2 Spanish talkers, 22 had English as their L1 and one L2 Spanish talker spoke Tagalog as their L1. L2 proficiency levels are available for most of the talkers from the RPD (63/70 or 90%) showing a concentration at the intermediate and advanced/near-native levels (French: beginner = 4, intermediate = 17, advanced/near-native = 24; Spanish: intermediate = 7, advanced/near-native = 15).

Table 2 provides an overview of the 21 variables that describe each of the 351 recordings. Of these 21 variables, eleven are grouping or identifying variables, seven are phonetic parameters, and three are information transmission variables. The grouping and identifying variables provide information about the talkers and recordings from the ALLSSTAR Corpus and Romance Phonetics Database that were included in the dataset.

The seven phonetic variables were automatically extracted from each recording using a published Praat script (de Jong & Wempe, 2009)^3^ that detects intensity peaks surrounded by intensity dips of at least 2 dB and that rise above a threshold that is determined as 25 dB below the 99^th^ quantile of the intensity maximum for the entire sound file. The 2 dB and 25 dB settings are adjustable defaults designed to minimize the influence of non-speech sound bursts in the intensity peak picking process. Furthermore, peaks that occur during unvoiced portions of the signal are excluded. The output of the Praat script provides the basis for calculating three measures of temporal structure: nsyll (number of acoustic syllables), SR (speech rate) and AR (articulation rate). SR is the rate of syllable production over the entire recording including pauses and other major disfluencies (nsyll/total duration), while AR is the rate of syllable production with silent pauses and other major disfluencies removed (nsyll/total duration minus pauses and disfluencies). By definition, for any given recording, AR is greater than SR. Because pausing and other disfluencies may be more prevalent in L2 speech than in L1 speech (e.g., see de Jong, 2016; Trouvain, Fauth & Möbius, 2016; Matzinger, Ritt & Fitch, 2020 and references therein), the magnitude of L2 versus L1 differences in SR will, if anything, be larger than L2 versus L1 differences in AR. In order to adopt a conservative stance, this study focuses on AR rather than SR. Any AR differences between L2 and L1 speech would likely be larger for SR. Average syllable duration is calculated based on AR (i.e., average syllable duration is equal to 1/AR).

The three information transmission variables were information density (ID), information rate (IR), and syllable reduction (LOSS). While articulation rate expresses the number of speech units (acoustic syllables) per unit of time, information density expresses the amount of information encoded in each speech unit. The relation between units of information (i.e., meaning) and speech units is highly complex, abstract, and involves a degree of non-compositionality that precludes a straightforward mapping. However, following an approach developed by researchers at the Laboratoire Dynamique Du Langage (CNRS / Université Lyon 2) (Pellegrino et al., 2011; Coupé et al., 2019), we can compare the relative information densities of various productions of a given text (i.e., for a fixed meaning). As discussed above, this prior work compared speech rate, information density, and information rate across languages with a focus on the syllable as the relevant speech unit. In the present study, we adopt this approach to compare the syllable information density for productions of a given text by L1 and L2 talkers within each of three languages, English, French, and Spanish – that is, within each language, we compare the number of acoustic syllables over which the information in the given text (i.e., the message of the DHR sentences or the NWS fable) is distributed for L1 versus L2 speech. For example, Talker A may exhibit a higher rate of syllable reduction for a given text than Talker B; in which case, Talker A’s production of the text has a higher information density than that of Talker B because Talker A’s production encodes the information of the text in fewer acoustic syllables than that of Talker B. The syllables of Talker A’s utterance would, on average, encode a greater proportion of the total information of the text than the syllables of Talker B’s production of the same text.

For the purposes of the present within-language comparisons, syllable information density, ID, for each talker, T, for each text, K, was expressed as the inverse of the number of acoustic syllables produced, as shown in (1) below,

(1) $${\text{ID}}_{\text{KT}}=\mathrm{avgInfoPer}{\text{Syl}}_{\text{KT}}=1/{\text{nSyll}}_{\text{KT}}$$

where nSyll_KT_ is the number of acoustic syllables in the current talker’s, T’s, production of text K. The interpretation of this value is the average proportion of information encoded per acoustic syllable. Relatively low information density (ID) values indicate that the current talker distributes the information contained in the text over a relatively large number of acoustic syllables each of which encodes a relatively low proportion of the total information content of the text. Conversely, relatively high information density (ID) values indicate production of relatively few acoustic syllables each of which encodes a relatively high proportion of the total information of the text.

The information rate, IR, for each recording was calculated based on the average acoustic syllable duration (i.e., the inverse of the number of acoustic syllables per second) and information density, ID (i.e., the average proportion of the total information of the text encoded per acoustic syllable), as in (2) below,

(2) $$\begin{array}{c} {\text{IR}}_{\text{KT}} \end{array}\begin{array}{c} =\\ = \end{array}\begin{array}{c} {\mathrm{avgInfoPerSec}}_{\text{KT}}={\text{ID}}_{\text{KT}}/{\text{avgSylDur}}_{\text{KT}}\\ \left(1/\text{nSy}{\|}_{\text{KT}}\right)/{\text{avgSylDur}}_{\text{KT}} \end{array}$$

where IR_KT_, ID_KT_, and avgSylDur_KT_ are the acoustic syllable information rate, information density, and average acoustic syllable duration for the current talker’s, T’s, production of text K, respectively. The information conveyed per second, IR, is thus the information encoded per acoustic syllable divided by the seconds per acoustic syllable, to yield the information conveyed per second. The units for IR are: (info/syl) / (sec/syl) = (info/syl) * (syl/sec) = info/sec.

A relatively high IR, can result from either a high information density (relatively few, information dense syllables for a given meaning), a fast articulation rate (short durations of acoustic syllables), or a combination of high ID and fast AR. Conversely, a relatively low IR can result from either a low information density (relatively many, information sparse syllables for a given meaning), a slow articulation rate (few syllables per second), or a combination of low ID and slow AR. As discussed in great depth in Pellegrino et al., 2011 and Coupé et al., 2019, across languages there appears to be a trade-off between information density and speech rate that imposes a limit on cross-language variation in information rate: languages with high information density tend to also exhibit slow speaking rates and vice versa.

In the present study, we compare information rate (IR) of L1 and L2 speech within each text (English DHR, English NWS, French NWS, and Spanish NWS). For the L2 recordings, the IR values might be expected to reflect either a compounding of ID and AR differences (if both ID and AR are lower for L2 than L1 speech) or a counteraction of ID and AR differences (if ID is higher but AR is lower for L2 than L1 speech). IR comparisons across L1 and L2 speech are therefore interpreted as indices of syllabic information transmission efficiency taking into account both duration and number of syllables produced for a given text.

Finally, syllable loss (LOSS) is a measure of syllable level phonetic reduction as expressed through the difference between the number of acoustic and orthographic syllables. Automatic measurement of speech rate based on acoustic syllable detection is an objective, purely signal driven technique that proceeds without reference to the linguistic content of the utterance, individual talker characteristics, or other parameters related to language-specific phonotactics. Due to connected speech processes that result in extensive phonetic variation (including, but not limited to, redundancy-driven reduction processes), acoustic syllables are not transparently related to the underlying abstract metrical and information bearing units of language production and perception. Specifically, the number of acoustic syllables produced for a given utterance will frequently differ from the sum of canonical, phonological syllables in the words that constitute the text of the utterance based on syllable counts in, for example, a pronunciation dictionary or as counted by L1 speakers in a metalinguistic syllable-counting task (e.g., by tapping out the number of syllables in a word). In order to relate acoustic syllables to orthographic syllables and to understand any observed variation across L1 and L2 speech in the information transmission variables that are based on acoustic syllables (i.e., information density, ID, and information rate, IR), we define syllable-level phonetic reduction, σ_LOSS_, as the number of acoustic syllables for a given utterance relative to the number of orthographic (i.e., phonological) syllables that constitute the linguistic structure of the utterance as in (3) below:

(3) $$\sigma_{\text{LOSS}}=\left(\sigma_{\text{a}}-\sigma_{\text{o}}\right)/\sigma_{\text{o}}$$

where σ_a_ is the number of acoustic syllables and σ_o_ is the number of orthographic syllables.

For the English recordings in the present study, the number of orthographic words and syllables was obtained using an English syllable counting function implemented in R (Kendall, 2013). This function applies a general rule for syllable counting based roughly on the number of orthographic vowels with adjustments for common orthographic deviations from the one-vowel-one-syllable rule. For example, a word’s syllable count is decreased if it contains ‘tia’ (e.g., ‘inertia’ has 3 syllables but 4 orthographic vowels) and a word’s syllable count is increased if it contains ‘ism’ (e.g., ‘schism’ has 2 syllables but 1 orthographic vowel). The function also specifies a list of exceptional cases to which the user can add any words that are not already included in this list of exceptions. For the DHR sentences, two words were added to the list of “special” 2-syllable words, “peaceful,” and “movement,” and two words were added to the list of “special” 3-syllable words, “entitled,” and “realized.” A close inspection of the counts returned by the function determined that the syllable counts for all other words in the English texts were acceptable with a conservative count for the few words (less than 1%) with ambiguous syllabification due to sonorants in coda position. These include ‘cruel’ (2 syllables), ‘prior’ (2 syllables), ‘realized’ (3 syllables), and ‘hours’ (2 syllables). It is therefore possible that for some speakers the rates of English syllable reduction were very slightly over-estimated. For the French and Spanish recordings, the number of orthographic words and syllables were hand counted and checked with L1 speakers. All orthographic word and syllable counts are shown in Table 3.

## 2. Results

**Fig. 1.**
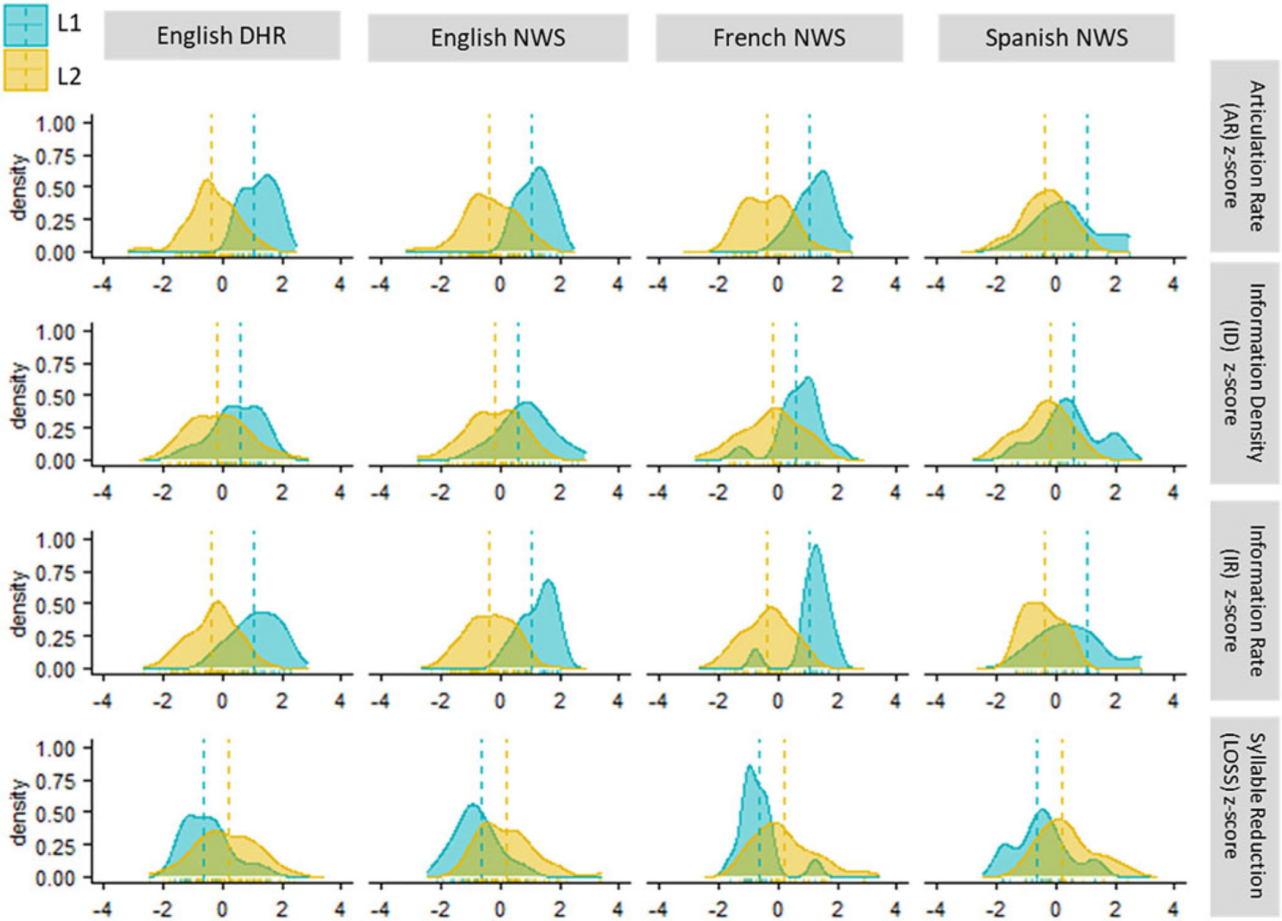
Density plots of articulation rate (AR), information density (ID), information rate (IR) and syllable reduction (LOSS) for the L1 and L2 groups within each recording text (English DHR, English NWS, French NWS, and Spanish NWS). All data are shown on z-transformed scales within their own distributions.

**Fig. 2.**
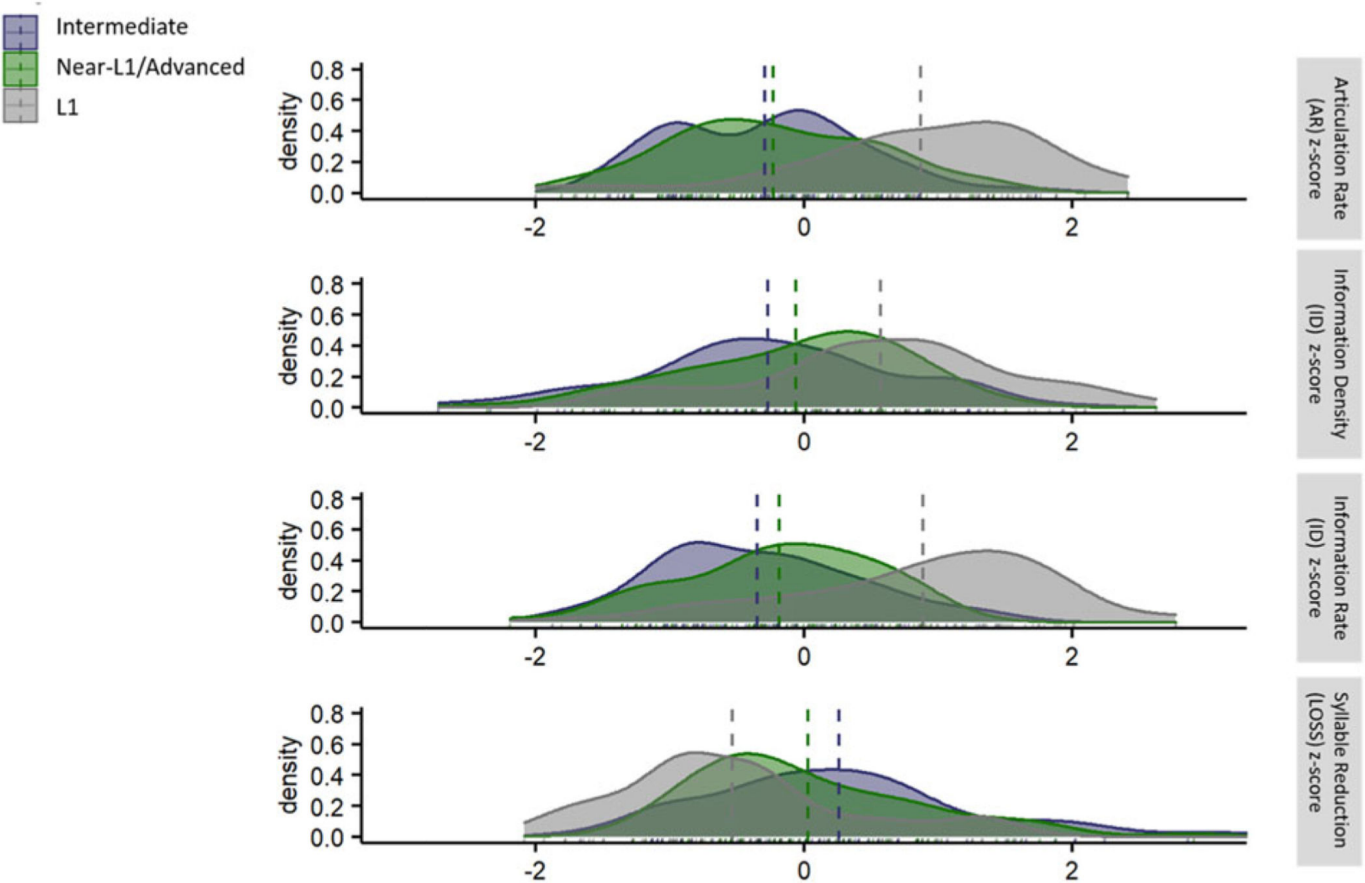
Density plots of articulation rate (AR), information density (ID), information rate (IR) and syllable reduction (LOSS) by proficiency group (L2 Intermediate, L2 Near-L1/Advanced, and L1) aggregated across texts and languages. All data are shown on z-transformed scales within their own distributions.

Table 4 shows summary statistics (mean and standard error of the mean) for six variables: (1) articulation rate (AR), (2) acoustic syllable duration, (3) number of acoustic syllables, (4) information density (ID), (5) information rate (IR), and (6) syllable reduction (LOSS). Data are shown for L1 speech and L2 speech for each of the four texts, English DHR, English NWS, French NWS, and Spanish NWS. Variables (2) and (3) are included for reference but are not included in visualizations (figures 1 and 2) or statistical modeling since they are directly derived from Variables (1) and (4), respectively. Variable (2), acoustic syllable duration is the inverse of variable (1), articulation rate. Variable (3), number of acoustic syllables is the inverse of variable (4), information density.

Figure 1 shows density plots for each of the four critical variables (AR, ID, IR, and LOSS) for each of the four texts (ENG_DHR, ENG_NWS, FRA_NWS and SPA_NWS). Each density plot compares the distributions for the L1 and L2 talkers. Since the texts varied substantially in terms of length (see Table 3), the data shown in these density plots were all z-transformed within their own distributions (i.e., within Text) allowing for a uniform horizontal scale for all frames in the composite figure.

Consistent with prior comparisons of articulation rate (AR) in L1 and L2 speech, the data in Table 4 and top row of Figure 1 show slower average articulation rates (fewer acoustic syllables per second) for L2 speech than for L1 speech. On average, the L2 articulation rates for the four texts are 85% (English DHR), 85% (English NWS), 84% (French NWS), and 95% (Spanish NWS) of their L1 counterparts. Accordingly, the average syllable durations for L2 speech are longer than for L1 speech; the average L2 syllable durations are 119% (English DHR), 118% (English NWS), 120% (French NWS), and 104% (Spanish NWS) of their L1 counterparts.

In addition to articulatory slowing (fewer syllables per second), L2 speech is also characterized by a consistent increase in the number of acoustic syllables produced relative to L1 speech (second row of Figure 1). For each of the four recording sets, the average number of L2 acoustic syllables is over 100% of the L1 counterpart: 105%, 110%, 107%, and 105% for English DHR, English NWS, French NWS, and Spanish NWS, respectively – that is, the number of acoustic syllables over which the information of the text (i.e., its intended meaning) is distributed is greater for L2 than for L1 speech, and consequently, the average proportion of information encoded per syllable (the average information density, ID) is lower for L2 than for L1 speech. The ID for L2 speech in each case is less than 100% of the L1 counterpart: 95% (English DHR), 90% (English NWS), 94% (French NWS), and 95% (Spanish NWS).

This combination of acoustic syllables that are relatively long in duration and relatively sparse in information content resulted in a substantially lower information rate for L2 speech relative to L1 speech (third row of Figure 1). The L2 average information rates (proportion of the total text meaning conveyed per second) are 81%, 77%, 78%, and 90% of their L1 counterparts for English DHR, English NWS, French NWS, and Spanish NWS, respectively. Thus, for all four texts, the slower articulation rate of L2 speech is compounded by the lower L2 syllable information density, to yield a substantially lower information rate for L2 speech than L1 speech – that is, the information (or, meaning) of a given text is encoded over a relatively large number of relatively long syllables with the consequence that the proportion of the text’s information that is conveyed per second is quite low for L2 speech as compared to L1 speech.

The final rows of Table 4 and Figure 1 provide a comparison of the rate of syllable reduction across L1 and L2 speech. These comparisons show that for all four texts, in both L1 and L2 speech the number of acoustic syllables in the speech signals generally falls below the number of orthographic syllables in the corresponding written texts – that is, almost all average syllable reduction values shown in Table 4 are negative (fewer acoustic than orthographic syllables). The only exception is for L2 French where the average reduction is slightly positive (indicating some syllable insertion). Critically, these average data show that for all texts there is more syllable reduction for L1 than for L2 speech. In other words, the low information density of L2 speech relative to L1 speech is due to extensive syllable-level reduction by L1 talkers rather than to ‘extra’ syllable insertion by L2 talkers.

Statistical modelling of the data was conducted in R (RStudio Version 1.3.959) using generalized linear mixed effects regression models (glmmTMB package). Separate analyses were conducted for each of the four critical dependent variables, articulation rate (AR), information density (ID), information rate (IR), and syllable reduction (LOSS). In each analysis, the effects of interest were the fixed effect of Group (L1 versus L2) and its interaction with Text (English DHR, English NWS, French NWS, and Spanish NWS). Forward contrast coding was applied to both categorical fixed factors, Group (2 levels) and Text, (4 levels). A random intercept for talker was included in the models. For all four dependent variables (AR, ID, IR, and LOSS), gaussian, beta, and gamma distributions were compared via the Akaike information criterion (AIC) resulting in selection (minimum AIC) of gaussian distributions for AR and LOSS, and gamma distributions for ID and IR. To assess the improvement in fit of the models with the interaction term (Talker*Group), log likelihood tests (anova) were run between the model with the interaction term and the model with only the additive term (Talker + Group, i.e., without the interaction term). For all four dependent variables, the model fit was significantly improved with addition of the interaction term. The model comparisons and predictors in the models with the interaction term are summarized in Tables 5 and 6, respectively.

Pairwise comparisons for the critical effect of interest (i.e., L1 versus L2 within each text) are shown in Table 7. For all four dependent variables (AR, ID, IR, and LOSS), these comparisons show significant differences between L1 and L2 talkers within all four texts (ENG_DHR, ENG_NWS, FRA_NWS, and SPA_NWS) with some variation in the magnitude of these differences thereby yielding the significant improvement in model fit with inclusion of the Group-by-Text interaction term as shown by the model comparisons in Table 5.

In order to gain some insight into a possible relation between L2 proficiency and the observed patterns of variation in AR, ID, IR and syllable reduction (LOSS), a subset of the data was examined by proficiency group. However, this post-hoc and unplanned division of the binary grouping variable (L1 versus L2) into more fine-grained proficiency-based sub-groups should be considered preliminary and suggestive rather than conclusive because there was no consistent and reliable proficiency assessment across the ALLSSTAR and RPD datasets, and proficiency data were not available for all talkers. The labels “Beginner,” “Intermediate,” and “Advanced/Near-L1” were intended as approximate groupings and the available data do not support identification of clear boundaries between these proficiency labels. Moreover, as noted above, no proficiency data was available for a substantial portion of the L2 talkers (approximately 17%), and the “Beginner” subgroup was very small (only 5 individuals across the full dataset). Notwithstanding these limitations, a subset of the data was examined in order to see if there was any indication that the observed L1-L2 differences in AR, ID, IR, and LOSS decrease with increasing L2 proficiency.

The sub-dataset for this proficiency-based examination excluded L2 talkers for whom L2 proficiency information was not available (n = 28). In addition, L2 talkers at the beginner level were excluded due to the small number of L2 talkers at that level of L2 proficiency (n = 5). Finally, to avoid analyses based on very small groups, the proficiency focused analyses were conducted on data that were aggregated across texts (i.e., across language groups). The sub-dataset therefore excluded data for the ENG-DHR text in order to avoid double-counting the individual talkers in the English group. The final proficiency dataset thus included data from 80.4% of the L2 talkers and 100% of the L1 talkers in the total dataset with the distribution across proficiency levels as follows: intermediate (n = 67), advanced/near-L1 (n = 68), and L1 (n = 59).

Density plots for each of the four critical variables by proficiency group are shown in Figure 2. Separate analyses were conducted for each of the four dependent variables, articulation rate (AR), information density (ID), information rate (IR), and syllable reduction (LOSS). In each analysis, the effect of interest was the fixed effect of Proficiency Group (Intermediate, advanced/near-L1, and L1). This 3-level factor was coded with the forward contrast scheme. For all four dependent variables, there was a significant effect of Proficiency Group (AR: F(191) = 74.6, p < .0001; ID: F(191) = 5.53, p < .005; IR: F(191) = 49.27, p < .0001; LOSS: F(191) = 27.55, p < .0001). Pairwise comparisons confirmed consistently significant differences for all four dependent variables between the L1 group and each of the L2 groups, intermediate (all p < .005) and advanced/near-L1 (all p < .04). The difference between the two L2 groups, intermediate and advanced/near-L1 was significant at the p < .0005 level for AR, IR, and LOSS; but the difference between these two L2 groups was not significant for ID. Thus, while this proficiency analysis should be viewed with caution, there is some suggestion that AR, ID, IR, and LOSS are all dynamic features of L2 speech and that the observed difference between L1 and L2 speech along these parameters may diminish with increasing experience with the target language.

## 3. Discussion

This study set out to identify a language-general acoustic marker of L1 versus L2 modes of speech production in terms of information theoretic parameters that have proved powerful for explaining intra-language variability in L1 speech. As such, this study represents a first step towards a broader goal of understanding information transmission via an L2 speech channel. The data showed that L1 and L2 productions of the same text within each of three typologically distinct languages (English, French, and Spanish) diverged along two phonetic parameters that define the temporal structure of the speech signal at the utterance level: articulation rate (the number of acoustic syllables per second) and information density (the number of acoustic syllables over which the information of the text is distributed). Specifically, in comparison to L1 speech, L2 speech was produced with a slower rate of articulation (fewer acoustic syllables per second) and a lower information density (more acoustic syllables for the given text). In combination, these two salient features of utterance-level speech timing each compounded the other such that the proportion of the total information of the text that was conveyed per second, the information transmission rate, was substantially lower for L2 than for L1 speech. Notably, both L1 and L2 speech generally involved production of substantially fewer acoustic syllables than orthographic syllables (as counted from the text scripts) indicating that the lower average information density for L2 speech is related to avoidance of phonetic reduction rather than a predisposition towards syllable insertion. As discussed in the introduction, English, French, and Spanish were well suited to this cross-language comparison due to their divergence along several relevant dimensions of sound structure including segment inventory size, consonant-to-vowel ratio, complexity of syllable structure, and several phonological processes that affect connected speech timing. The convergent pattern of lower information transmission rate for L2 than L1 speech across these languages suggests that this may be a language-general distinguishing feature of L2 versus L1 speech.

This investigation was inspired by prior work supporting the claim that a significant portion of surface variation within and across languages can be attributed to a principle of efficiency of information encoding and transmission (see Gibson, Futrell, Piantadosi, Dautriche, Mahowald, Bergen & Levy, 2019 for an overview of this approach). Under this view, a major source of intra-language variation across levels of speech and language structure is the modulation of density of information encoding through structural and/or phonetic reduction in accordance with variation in contextually-determined suprisal, or predictability. Moreover, while syllabic rate (number of syllables per second) and syllabic information density (number of syllables for a given meaning) vary substantially across languages – due to structural differences including phonotactics and phoneme inventory – cross-language variation in communicative efficiency is constrained via a trade-off between articulation rate and information density (Pellegrino et al., 2011; Coupé et al., 2019). Within this conceptual framework, which emphasizes optimization of communicative efficiency, and building on this empirical approach, which operationalizes information rate in terms of automatically measurable phonetic units, the present study establishes a solid empirical base from which we can now speculate on the underlying causes and communicative consequences of the distinct L1 versus L2 information encoding and transmission profiles.

From the perspective of the message encoder (i.e., the talker), both the slower rate and the increased number of acoustic syllables in L2 speech may originate from general features of L2 speech production regardless of the particular L2 being spoken and regardless of the talker’s L1. The slower articulation rate is likely related to slower processes at multiple levels, including lexical retrieval, production planning, and speech articulation (e.g., see Broos, Duyck & Hartsuiker, 2018). Identification of the underlying mechanisms for this general slowing of L2 production is an active topic of current research with proposals including L1-L2 interactions (i.e., bi-directional competition and/or interference) and L1 versus L2 frequency-of-usage differences (for reviews see Kroll & Gollan, 2014; Runnqvist, Strijkers & Costa, 2014). Regardless of the cognitive, linguistic, and/or articulatory differences between L2 and L1 speech production, the present data are consistent with prior demonstrations that the encoding of a given message in L2 speech typically results in a speech signal with a relatively slow average syllable-level temporal modulation rate (see also Bradlow et al., 2017; Baese-Berk & Morrill, 2015; Guion et al., 2000). For the recordings in this study, the overall average L1 and L2 articulation rates were 4.6–4.9 cycles per second and 3.9–4.4 cycles per second, respectively. For both L1 and L2 speech, these modulation rates tend towards the low end of the theta neural oscillation band of approximately 4–8 Hz, which has received considerable attention as one of a series of correspondences between speech unit durations and neural oscillator frequency ranges. Gamma (>40 Hz) and beta (15–30 Hz) oscillations correspond to phonetic features with 20–50 ms durations, theta (4–8 Hz) oscillations correspond to syllables and words with ∼ 250 ms durations, and delta oscillations (<3 Hz) correspond to phrases with 500–2000 ms durations. These correspondences have inspired theories linking speech perception and neurophysiology (e.g., Poeppel, 2003; Ahissar & Ahissar, 2005; Ghitza & Greenberg, 2009; Ghitza, 2011, 2012; Giraud & Poeppel, 2012; Peelle & Davis, 2012; but see Cummins, 2012 for a more skeptical view of this approach). The correspondence between theta band neural oscillations and syllable rate, in particular, has inspired the proposal of “theta-syllables” (Ghitza, 2013) whose definition (“a theta-cycle long speech segment located between two successive vocalic nuclei”) closely matches that of the acoustic syllable of the present study. Importantly, while the general slowing of L2 speech relative to L1 speech is quite consistent across individuals and languages, it remains within the theta range of 3.5–7.5 Hz. Thus, from an auditory processing perspective, the magnitude of the reduced articulation rate for L2 speech compared to L1 speech, though quite reliable, is probably not large enough to alter basic auditory processing that may be driven by theta-band neural oscillations.

While the processes of message encoding in L2 speech result in subtle yet reliable changes to the temporal modulation rate of the L2 speech signal, the concurrent reduced prevalence of phonetic reduction, and consequent lowering of syllable information density, alters the mapping between acoustic content and linguistic representations. Under the view that phonetic reduction is driven by a principle of uniform information density, or smooth signal redundancy, we can envision several mutually compatible sources of a lack of phonetic reduction in L2 speech. First, it is possible that during L2 speech production, optimization of information transfer through redundancy-related reduction is down-weighted in favor of resource allocation to other processes of language and speech production (lexical selection, production planning, and articulation) which are less practiced and therefore more resource demanding in L2 compared to L1 speech. This possibility is consistent with the notion of phonetic reduction (and its counterpart, phonetic strengthening) as an active, and therefore resource demanding, process of information transmission optimization that talkers implement during message encoding.

A second possibility is that connected speech in L1 and L2 involve similar reduction-related optimization processes, but the language models from which probabilities of occurrence are derived differ due to different contexts in which the language was learned and is used. Later onset and shorter duration are defining features of L2 versus L1 acquisition, and in contrast to the typical implicit L1 learning context, the L2 learning context often involves explicit instruction with an emphasis on written forms (see Lupyan & Dale, 2010 for discussion of L1 versus L2 learning contexts). Furthermore, L2 learning inevitably involves interaction with the L1 – which, depending on the typological relationship between the structures of the particular L1 and L2, may be facilitatory, neutral, or inhibitory. All of these features of L2 learning would likely result in distinct L1 and L2 language models from which probabilities of occurrence are derived, resulting in different patterns of redundancy-driven phonetic reduction. Evidence that different L1 and L2 reduction patterns reflect different underlying language models rather than differences in the reduction processes per se come from studies that have demonstrated similar degrees of later-mention reduction in L2 and L1 speech (e.g., Baker et al., 2011; Gustafson & Goldrick, 2018; Spilková, 2014; Oh & Lee, 2016; and Li et al., 2018). All of these studies showed that the second or later mention of a word in a discourse (Baker et al., 2011; Gustafson & Goldrick, 2018; Spilková, 2014; Oh & Lee, 2016; and Li et al., 2018) or in a sequence of experimental trials (Gustafson & Goldrick, 2018) was shortened relative to its first mention by a similar amount in L2 as in L1 speech suggesting similar tracking of local, discourse-level probability for L1 and L2 speech. These studies thus support the view that distinct L1 versus L2 reduction patterns are related to their distinct contexts of learning and experience rather than to down- or up-weighting optimization of information transfer depending on resource availability.

The complexity of the language models over which surprisal/redundancy is estimated has been highlighted in several detailed studies of redundancy-driven reduction in L1 speech (e.g., Aylett & Turk, 2004, 2006; Bell et al., 2009; Cohen Priva, 2015; Gahl et al., 2012; Johnson, 2004; Jurafsky et al., 2001; Turk, 2010). Using large corpora of spontaneous speech with linguistic annotations and extensive automatic phonetic measurements, these studies have demonstrated a tight relationship between probability of occurrence given the context and various measures of phonetic reduction in both the temporal and spectral domains. The Smooth Signal-Redundancy Hypothesis (Aylett & Turk, 2004, 2006; Turk, 2010) has proposed that prosodic prominence and boundary structure mediate the implementation of redundancy-driven variation in articulatory strength, probably in combination with direct temporal and spectral adjustment. Importantly, effective implementation of redundancy-related reduction, whether through prosodic restructuring or through direct temporal and spectral adjustment of syllables and segments, depends on long-term knowledge and frequent usage of the language in varying contexts. It thus follows naturally that the distinct learning and usage patterns of L2 and L1 speech will strongly impact their respective patterns of redundancy-related reduction.

Furthermore, L2 learning often relies heavily on the written medium which could lead to prioritization of production targets based on orthographic forms with fully specified syllables over frequently used reduced forms. For example, the word “*traveler*” in the English NWS passage is frequently pronounced with two syllables in L1 English. However, in L2 English, this word is frequently pronounced with three syllables. The unstressed middle syllable of the orthographic form is often fully articulated in L2 speech but reduced in L1 speech. Similarly, in the French NWS passage, the word “*enveloppé*” may be pronounced with four full syllables in L2 French but with a reduced second syllable in L1 French, and in the Spanish NWS passage, the word “*viento*” may be pronounced with three syllables in L2 Spanish but with two syllables in L1 Spanish. These L1 reductions may reflect redundancy, or predictability, based on lexical frequency, morphological composition, phonotactic sequencing, and/ discourse context. In contrast, for L2 talkers, their learning context and usage-based experiences may prioritize the orthographically-driven pronunciation resulting in production of “extra” acoustic syllables, thereby lowering the L2 speech information density and transmission rate.

From the perspective of the listener at the receiver’s end of the speech transmission chain, what might be the consequences for message decoding of the slower information transmission rate of L2 compared to L1 speech? Studies of L2 speech recognition by L1 listeners have demonstrated highly generalized improvement in L2 speech recognition accuracy following repeated exposure to L2 speech samples (e.g., Bradlow & Bent, 2008; Baese-Berk, Bradlow & Wright, 2013; Sidaras, Alexander & Nygaard, 2009; Tzeng, Alexander, Sidaras & Nygaard, 2016; Xie & Myers, 2017; Xie, Weatherholtz, Bainton, Rowe, Burchill, Liu & Jaeger, 2018; Alexander & Nygaard, 2019), suggesting that listeners can learn new message decoding routines in response to novel message encoding features. However, even with effective perceptual adaptation to L2 speech in terms of improved intelligibility (i.e., improved word recognition accuracy), the slow information transmission rate of L2 speech signals would presumably limit its communicative efficiency in terms of processing time.

The divergence of intelligibility (i.e., recognition accuracy) and communicative efficiency (i.e., processing time) is also illustrated by the contrast between L2 speech and “clear” speech, a speech style that talkers often adopt when they are aware of a speech communication barrier for the listener due to, for example, a hearing impairment or environmental noise (for reviews of clear speech research, see Krause & Braida, 2004; Smiljanic & Bradlow, 2009; Smiljanic, 2021). In comparison to “plain” (or “conversational”) L1 speech, both clear speech and L2 speech are characterized by slow articulation rates (fewer syllables/second) and avoidance of phonetic reduction (i.e., lower information density). However, while clear speech is associated with enhanced intelligibility, L2 speech is associated with reduced intelligibility. Crucially, the phonetic encoding adjustments of clear speech are driven by the principle of communicative efficiency that is at the heart of the Smooth Signal Redundancy and the Uniform Information Density hypotheses. Under conditions of reduced predictability/redundancy due to environmental or listener-related degradation of the communication channel, talkers adopt a mode of message encoding that involves phonetic enhancement to compensate for lost or degraded information in the transmission channel. Thus, while both L2 speech and clear speech involve a combination of slow articulation rate and low information density to yield a low information transmission rate, they differ dramatically with respect to their impact at the receiver’s end of the information transmission system. The slow articulation rate and low information density of clear speech production are in direct response to the presence of environmental or receiver-related noise, and therefore effectively enhance intelligibility and communicative efficiency in the compromised speech communication context – that is, clear speech adjustments at the transmission end are designed to counteract impedance to information flow in the transmission channel or at the receiver’s end of the communication system. In contrast, the slow articulation rate and low information density of L2 speech production are internal to the message encoder (the talker) and therefore introduce an element of potential disruption to the smooth and efficient flow of information across the speech communication system as it extends from the transmitter (talker) through the communication channel (speech signal) to the receiver (listener).

The focus of the present study was on the compounding effects of slow articulation rate (few syllables/second) and low information density (many syllables for a given message) to yield a slow information rate (low proportion of total information conveyed per second) for L2 speech compared to L1 speech. A key feature of the data presented in this study is that the L1 and L2 speech recordings within each of the three languages (English, French, and Spanish) were readings of a given text. Thus, the within-language comparisons of L1 versus L2 speech were controlled for intended meaning, allowing us to view the rate and number of acoustic syllables produced in relation to the encoding of a fixed meaning in L1 versus in L2 speech. However, this dataset was rather limited in terms of statistical modelling of phonetic reduction as a function of surprisal, or probability, of linguistic forms in various contexts. Detailed analyses with large corpora of L2 speech would allow for direct comparison of the relationship between probability of occurrence given the context (i.e., redundancy, or conversely, surprisal) and phonetic reduction in L2 and L1 speech. This would, in turn, allow for deeper insight into the causes and consequences of the distinct temporal modulation pattern of L2 speech across various languages and for hypothesis-driven testing of alternative explanations for the observed differences between L1 and L2 information encoding and transmission

## 4. Conclusion

This study provides cross-language evidence that the characteristically slow rate of L2 speech (low number of acoustic syllables per second) combines with a tendency towards fully articulated rather than phonetically reduced forms (i.e., low information density) to produce an L2 speech style with a very low information transmission rate. This compounding of slow articulation rate with low information density in L2 speech contrasts with the efficiency-driven trade-off between speech reduction and information density that characterizes L1 speech. Future research with large-scale, multi-lingual, multi-talker corpora of L1 and L2 speech under natural, conversational conditions and in a wide range of languages are needed to gain further insight into the dynamics of communicative efficiency via L2 speech channels.

## Figures and Tables

**Table 1.** Overview of recordings. See text for detailed explanations.

Source		Northwestern University ALLSSTAR Corpus (Total of 248 recordings)		University of Toronto Romance Phonetics Database (Total of 103 recordings)			
Text		DHR Sentences and NWS Passage		NWS Passage			
Language		English		French		Spanish	
Talker Group		L1	L2	L1	L2	L1	L2

Talkers (N)		26	98	14	47	19	23

Average age (years)		20	25	31	27	29	31

Female / Male		14 F	36 F	8 F	37 F	13 F	10 F
	
		12 M	62 M	6 M	10 M	6 M	13 M

Average age of L2 onset (range)		--	9.4 yrs (0–28)*	--	9.9 yrs (3–29)	--	18 yrs (0–32)*

L2 Proficiency	Beginner (5)	--	1	--	4	--	0
	
	Intermediate (67)	--	43	--	17	--	7
	
	Advanced/Near-L1 (68)	--	29	--	24	--	15
	
	Not available (28)	--	25	--	2	--	1

* Only two talkers reported an age of L2 acquisiton of 0 years, one with Cantonese as the L1 (ALLSSTAR Corpus, L2 English) and one with Tagalog as the L1 (RPD Corpus, L2 Spanish).

**Table 2.** Overview of variables. See text for detailed explanations.

Grouping and identifying variables (see text for further explanation)			
Variable name			Description
1. corpus			ALL (ALLSSTAR) or RPD (Romance Phonetic Database)
2. talkerCode			Unique identifier for each talker (alphanumeric for RPD)
3. talkerNum			Unique numerical identifier for each talker
4. m_f			Male or female
5. l1			Talker’s L1
6. textLang			English, French, or Spanish
7. text			NWS or DHR
8. group			L1 or L2
9. age			Age in years at the time of recording (self-reported)
10. targetProficiency			Beginner, Intermediate, Advanced/Near-L1, L1
11. aoaL2			Age of L2 acquisition (self-reported)
Phonetic variables (see text for further explanation)			
Variable name		Unit	Description
1. nsyll		number	number of acoustic syllables
2. npause		number	number of silent pauses
3. dur		seconds	utterance duration
4. phonationTime		seconds	phonation time (sum of ‘sounding’ segment durations)
5. speechRate		syll/sec	nsyll per dur (includes pauses)
6. articulationRate (AR)		syll/sec	nsyll per phonationTime (excludes pauses)
7. avgSylDur		sec/syll	average syllable duration (1/AR)
Information transmission variables (see text for further explanation)			
Variable name	Description		
1. avgInfoPerSyl (ID)	1/nSyll	syllable information density (ID), proportion of total text information encoded per acoustic syllable	
2. avgInfoPerSec (IR)	avgSylDur / ID	syllable information rate (IR), proportion of total text information conveyed per second	
3. syllRed (LOSS)	(nsyll-ortho) / ortho	syllable reduction (LOSS), number of acoustic syllables relative to number of orthographic syllables	

**Table 3.** Number of sentences, words, and orthographic (i.e., phonological) syllables in the NWS passages and DHR sentences.

	Sentences	Words	Orthographic (phonological) Syllables
English NWS passage	5	113	144
English DHR sentences	20	319	522
French NWS passage	6	108	165
Spanish NWS passage	5	99	184

**Table 4.** Articulation rate (AR), acoustic syllable duration, number of acoustic syllables, information density (ID), information rate (IR), and acoustic syllable reduction (LOSS) by talker group (L1 versus L2) and by recording text (English DHR, English NWS, French NWS, and Spanish NWS). Data shown are means with standard error of the mean in parentheses. See text for additional explanation for each variable.

Language	English				French		Spanish	
Text	DHR Sentences		NWS Passage		NWS Passage		NWS Passage	
Group	L1	L2	L1	L2	L1	L2	L1	L2
N	26	98	26	98	14	47	19	23

AR (acoustic syllables/second)	4.94	4.18	4.67	3.97	4.95	4.07	4.74	4.43
	(0.05)	(0.04)	(0.05)	(0.04)	(0.09)	(0.06)	(0.11)	(0.07)

Acoustic syllable duration (seconds)	0.203	0.242	0.215	0.254	0.203	0.248	0.213	0.227
	(0.002)	(0.003)	(0.002)	(0.003)	(0.004)	(0.004)	(0.005)	(0.004)

Number of acoustic syllables	459	483	126	139	150	169	161	176
	(5.9)	(3.9)	(1.9)	(1.3)	(3.9)	(3.2)	(3.6)	(3.3)

ID (%) (info/acoustic syll)	0.22	0.21	0.80	0.72	0.67	0.60	0.63	0.57
	(0.003)	(0.002)	(0.012)	(0.007)	(0.015)	(0.011)	(0.014)	(0.010)

IR (%) (info/second)	1.08	0.87	3.74	2.88	3.32	2.46	2.98	2.54
	(0.022)	(0.012)	(0.060)	(0.043)	(0.096)	(0.065)	(0.122)	(0.061)

Syll. reduction (%) (acoust. vs. ortho)	−12.1	−7.5	−12.7	−3.3	−9.0	2.3	−12.3	−4.5
	(1.1)	(0.7)	(1.3)	(0.9)	(2.3)	(1.9)	(2.0)	(1.8)

**Table 5.** Summary of comparisons between models with and without the Group-by-Text interactive term. In all cases the interactive model was a significantly better fit (lower AIC) than the additive model.

	Chi-squared	df	p
AR	14.78	3	<.003 (**)
ID	16.65	3	<.001 (**)
IR	15.93	3	<.002 (**)
LOSS	15.72	3	<.002 (**)

**Table 6.** Summaries of the best fit models with the Group by Text interactive terms. The referent category in all models is ENG_DHR and L1 for the Text and L1 factors, respectively.

	Predictors	Estimates	std. Error	Z value	Pr(>|z|)
AR Gaussian (identity)	(Intercept)	4.49	0.03	158.44	<0.0001 (***)
	Text [ENG_NWS]	0.24	0.02	9.55	<0.0001 (***)
	Text [FRA_NWS]	−0.19	0.07	−2.68	0.007 (**)
	Text [SPA_NWS]	−0.07	0.08	−0.90	0.368
	Group [L2]	0.66	0.06	11.69	<0.0001 (***)
	Text [ENG_NWS] * Group [L2]	0.07	0.05	1.39	0.165
	Text [FRA_NWS] * Group [L2]	−0.18	0.14	−1.24	0.214
	Text [SPA_NWS] * Group [L2]	0.56	0.16	3.44	<0.0001 (***)
	Marginal R^2^ / Conditional R^2^	0.439 / 0.898			
ID Gamma (log)	(Intercept)	−5.30	0.01	−758.5	<0.0001 (***)
	Text [ENG_NWS]	−1.27	0.01	−195.6	<0.0001 (***)
	Text [FRA_NWS]	0.18	0.02	10.5	<0.0001 (***)
	Text [SPA_NWS]	0.06	0.02	2.8	<0.005 (**)
	Group [L2]	0.09	0.01	6.3	<0.0001 (***)
	Text [ENG_NWS] * Group [L2]	−0.05	0.01	−4.1	<0.0001 (***)
	Text [FRA_NWS] * Group [L2]	−0.01	0.03	−0.3	0.75
	Text [SPA_NWS] * Group [L2]	0.03	0.04	0.7	0.51
	Marginal R^2^ / Conditional R^2^	0.974 / 0.995			
IR Gamma (log)	(Intercept)	−3.81	0.01	−336.2	<0.0001 (***)
	Text [ENG_NWS]	−1.22	0.01	−168.5	<0.0001 (***)
	Text [FRA_NWS]	0.14	0.03	5.1	<0.0001 (***)
	Text [SPA_NWS]	0.04	0.03	1.2	0.242
	Group [L2]	0.24	0.02	10.5	<0.0001 (***)
	Text [ENG_NWS] * Group [L2]	−0.05	0.01	−3.2	<0.005 (**)
	Text [FRA_NWS] * Group [L2]	−0.04	0.06	−0.8	0.4522
	Text [SPA_NWS] * Group [L2]	0.16	0.06	2.4	<0.02 (*)
	Marginal R^2^ / Conditional R^2^	0.935 / 0.994			
LOSS Gaussian (identity)	(Intercept)	−0.07	0.01	−10.66	<0.0001 (***)
	Text [ENG_NWS]	−0.02	0.01	−2.81	<0.01 (**)
	Text [FRA_NWS]	−0.05	0.02	−2.72	<0.01 (**)
	Text [SPA_NWS]	0.05	0.02	2.56	<0.02 (*)
	Group [L2]	−0.08	0.01	−5.99	<0.0001 (***)
	Text [ENG_NWS] * Group [L2]	0.05	0.01	3.87	<0.0001 (***)
	Text [FRA_NWS] * Group [L2]	0.02	0.03	0.52	0.6
	Text [SPA_NWS] * Group [L2]	−0.03	0.04	−0.86	0.39
	Marginal R^2^ / Conditional R^2^	0.199 / 0.841			

**Table 7.** Pairwise comparisons of the estimated means between L1 and L2 within each text.

L1 vs L2		Estimate	SE	df	t ratio	p value
AR	ENG_DHR	0.77	0.08	341	9.27	<.0001 (***)
	ENG_NWS	0.70	0.08	341	8.44	<.0001 (***)
	FRA_NWS	0.87	0.11	341	7.65	<.0001 (***)
	SPA_NWS	0.31	0.12	341	2.69	0.01 (*)
ID	ENG_DHR	0.05	0.02	341	2.40	0.02 (*)
	ENG_NWS	0.10	0.02	341	4.98	<.0001 (***)
	FRA_NWS	0.11	0.03	341	4.00	0.0001 (***)
	SPA_NWS	0.09	0.03	341	3.00	0.01 (*)
IR	ENG_DHR	0.22	0.03	341	6.75	<.0001 (***)
	ENG_NWS	0.27	0.03	341	8.18	<.0001 (***)
	FRA_NWS	0.31	0.05	341	6.85	<.0001 (***)
	SPA_NWS	0.15	0.05	341	3.33	0.001 (**)
LOSS	ENG_DHR	−0.05	0.02	341	−2.24	<0.03 (*)
	ENG_NWS	−0.09	0.02	341	−4.69	<.0001 (***)
	FRA_NWS	−0.11	0.03	341	−4.04	0.0001 (***)
	SPA_NWS	−0.08	0.03	341	−2.76	<0.01 (*)

## Footnotes

1 Note that, while the first-acquired language is usually the dominant language (i.e., exposure to the L1 is usually both early and extended), many bilingual adults exhibit a pattern of “switched dominance” language learning – that is, for many bilingual adults (often known as “heritage speakers”), the second-acquired language, the L2, is the dominant language due to extended exposure for the L2 in contrast to early but limited exposure to the L1. In the present paper, L2 should be understood as both the second-acquired and the non-dominant (i.e., the later and less exposed) language.

2 For review and extensive discussion of these accounts, as well as a nuanced analysis of the multiple factors that modulate phonetic reduction, see Clopper and Turnbull, 2018.

3 In order to reduce the influence of artefacts in the detection of acoustic syllables, all files were checked for spurious high-amplitude bursts (a noise burst from the end of one RPD file was removed during this process), and the root-mean-square intensity (RMS) of all files was equalized before running the Praat script. This step ensured equivalent application of the syllable detecting procedure, without adjustment to the default settings, to all speech files in both L1 and L2 speech and in all three languages.
